# A finite element model of the lower limb during stance phase of gait cycle including the muscle forces

**DOI:** 10.1186/s12938-017-0428-6

**Published:** 2017-12-07

**Authors:** Arnaud Diffo Kaze, Stefan Maas, Pierre-Jean Arnoux, Claude Wolf, Dietrich Pape

**Affiliations:** 1 Faculty of Science, Technology and Communication, University of Luxembourg, 6 Rue R. Coudenhove-Kalergi, 1359 Luxembourg, Luxembourg; 2 Department of Orthopedic Surgery, Centre Hospitalier de Luxembourg, 76 Rue d’Eich, 1460 Luxembourg, Luxembourg; 3 Sports Medicine Research Laboratory, Public Research Centre for Health, Luxembourg, Centre Médical de La Fondation Norbert Metz, 76 Rue d’Eich, 1460 Luxembourg, Luxembourg; 4 Cartilage Net of the Greater Region, 66421 Homburg/Saar, Germany; 5 Laboratoire de Biomécanique Appliquée, UMRT24 IFSTTAR-Université de la Méditerranée, Boulevard Pierre Dramard, 13916 Marseille Cedex 20, France

**Keywords:** Finite element, Musculoskeletal model, Rigid body, Lower limb, Muscle forces, Stance phase

## Abstract

**Background:**

Results of finite element (FE) analyses can give insight into musculoskeletal diseases if physiological boundary conditions, which include the muscle forces during specific activities of daily life, are considered in the FE modelling. So far, many simplifications of the boundary conditions are currently made. This study presents an approach for FE modelling of the lower limb for which muscle forces were included.

**Methods:**

The stance phase of normal gait was simulated. Muscle forces were calculated using a musculoskeletal rigid body (RB) model of the human body, and were subsequently applied to a FE model of the lower limb. It was shown that the inertial forces are negligible during the stance phase of normal gait. The contact surfaces between the parts within the knee were modelled as bonded. Weak springs were attached to the distal tibia for numerical reasons.

**Results:**

Hip joint reaction forces from the RB model and those from the FE model were similar in magnitude with relative differences less than 16%. The forces of the weak spring were negligible compared to the applied muscle forces. The maximal strain was 0.23% in the proximal region of the femoral diaphysis and 1.7% in the contact zone between the tibia and the fibula.

**Conclusions:**

The presented approach based on FE modelling by including muscle forces from inverse dynamic analysis of musculoskeletal RB model can be used to perform analyses of the lower limb with very realistic boundary conditions. In the present form, this model can be used to better understand the loading, stresses and strains of bones in the knee area and hence to analyse osteotomy fixation devices.

## Background

The biomechanics and the finite element (FE) analysis of the knee joint provide observations that are useful for clinical diagnoses of knee joint diseases. The FE method that is well established in the domain of biomechanics is used to capture tissue responses to external loads such as strains and stresses. For this purpose, the tissues are modelled as deformable bodies. The FE method represents an important tool for the design of knee joint prostheses and implants. It is therefore essential to consider realistic loading of the knee joint during the analysis as well as biomechanical testing as indicated by Brinkmann et al. [1]. The existing forces within the knee joint result from the combination of muscular forces, inertial forces, weight and ground reaction forces [2, 3]. This means that one should consider all these forces when modelling the knee joint. But many simplifications are made in models from the literature, essentially in order to reduce the complexity of the problem. For example, restricting the loading condition to compressive loads while the chosen knee flexion angle is kept constant [4–7]. Hao et al. [6] investigated the contact behaviour of the tibiofemoral joint by applying a compressive load on the knee joint while the knee flexion angle was kept constant at about 25°. Other authors considered compressive loads in their studies with knee flexion angle of 0° [4, 5, 7]. All the previously cited papers considered a single position of the lower limb and reduced the femur to its distal part and the tibia to its proximal part. Simulating more positions of the knee joint to replicate a normal human daily activity, like slow walking, and considering muscle forces, as indicated in the present study, would be more realistic and would give a more accurate insight into the knee biomechanics. Muscle forces are not measurable in vivo, but contact forces in the joints are measurable by means of telemetric instrumentation [8–12]. Although these joint contact forces are quantitatively different from one author to another, they are generally used to validate musculoskeletal models, which are used to predict muscle forces [13–16]. Kutzner et al. [11] reported a maximal difference of 100% BW between the resultants of the knee joint forces measured during walking in five different subjects. The musculoskeletal models are rigid body (RB) models [13–15] or coupled RB/deformable models. For the latter soft tissues within the joints are often modelled as deformable bodies, keeping the bones rigid as it is the case for musculoskeletal RB models [16–22]. Kiapour et al. [19, 20] applied knee abduction and internal tibia rotation moments under various knee flexion angles while taking into account the muscle actions as uniaxial elements. In the models of Kiapour and colleagues the muscles were passive and not creating the movement, but resisting the imposed moments. Considering the muscles as generators of movement would be more consistent with reality. Adouni et al. [21, 22] made an iterative musculoskeletal FE model of the lower limb in order to investigate the cartilage stresses during the stance phase and predict muscle forces. They considered the bones as rigid bodies. Their model was driven by kinematics and kinetics data collected during gait and they considered the actions of muscles by modelling them as uniaxial elements. The aim of this study is to present a different approach, which was used in order to make a FE model of the lower limb. Muscle forces were included as loading conditions and the bones were modelled as deformable bodies. The muscle forces were determined by means of a musculoskeletal RB model. Such a FE model can be used to analyse the performance of high tibial osteotomy (HTO) fixation devices.

## Methods

### Used musculoskeletal model

The forces of the muscles acting in the lower limb were predicted using a musculoskeletal rigid body (RB) model of the human body, and were subsequently applied to a FE model of the lower limb. The stance phase of normal gait was considered and simulated. The model “Gaitfullbody”, which is present in the model repository of the musculoskeletal modelling software AnyBody version 6.0 [23], was used to predict the muscle forces. The muscle prediction in AnyBody is based on the inverse dynamics method [24–26]. The min/max optimization criterion was used for muscle recruitment in the AnyBody modelling environment and is described elsewhere [27, 28]. The model “GaitFullbody” considers the normal gait of a person with a mass of about 62 kg and a height of 1.62 m. This model is derived from previous musculoskeletal models that have already been validated. The validation of the previous musculoskeletal models was made by comparing the predicted hip joint forces to the measured joint forces [13–15]. The knee joint being of interest for the present model, the experimental measured knee contact forces from the works of Bergmann et al. (file K7L_280710_1_28P from database OrthoLoad [12]) were compared to the knee joint forces of the “Gaitfullbody” model. The model was considered as valid and used to predict the muscle forces acting in the lower limb during normal gait, which were subsequently applied to the FE model.

### Muscle forces applied to the FE models

**Fig. 1:**
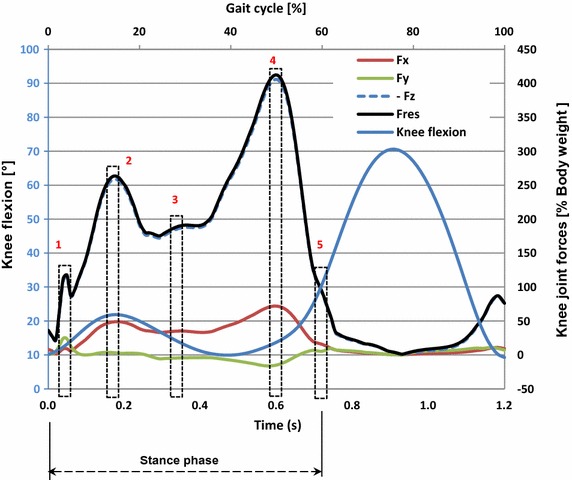
The 5 analysed positions: components of the knee joint contact forces and knee joint flexion angle during the gait cycle

The forces due to the acceleration of the thigh, the leg and the foot during, stance phase (Table 1), are negligible compared to the ground reaction forces (GRF) and the maximal muscle forces. For simplification purposes, inertial effects were ignored and static analyses were performed. Five load configurations representing five instants of the gait were selected (Fig. 1) in order to simulate the stance phase. They corresponded to the beginning (position 1) and end (position 5) of the stance phase, and the extrema of the knee joint force (positions 2, 3 and 4).

**Table 1:** Inertial forces of the lower limb in the selected five positions stance phase

	Thigh (m = 6.22 kg)				Leg and foot (m = 3.8 kg)			
	ma_x_ (N)	ma_y_ (N)	ma_z_ (N)	ma (N)	ma_x_ (N)	ma_y_ (N)	ma_z_ (N)	ma (N)
Position 1	1	24	8	26	17	13	− 3	22
Position 2	− 12	− 4	− 7	15	− 41	− 10	9	43
Position 3	− 3	− 11	− 2	12	4	− 3	− 4	7
Position 4	12	9	− 7	16	16	− 1	− 7	18
Position 5	− 15	8	2	17	30	− 8	2	32

The inertial forces were calculated as the product of the mass and the acceleration of the segment during the stance phase

The muscles of the foot and those that span only the ankle joint were not considered. But 29 muscles of the lower limb that bridge the hip and the knee joints were selected to be considered in the modelling of the lower limb: the gluteal muscles (gluteus maximus, medius and minimus), the iliopsoas (iliacus), the piriformis, the pectineus, the obturators internus and externus, the gemelli inferior and superior, the quadratus femoris, the adductors (adductor brevis, longus and magnus), the tensor fasciae latae, the sartorius, the gracilis, the long and short heads of the biceps femoris, the quadriceps femoris (rectus femoris, vastus intermedius, vastus lateralis and vastus medialis), the popliteus, the plantaris, the medial and the lateral head of the gastrocnemius.

Since the insertions or the origins of some of these muscles are relatively large surfaces, those muscles are subdivided into two or more subdivisions in the musculoskeletal model. The actions of the 29 selected muscles of the lower limb are modelled with 122 muscle forces in the musculoskeletal model. The 122 muscle forces were recombined into 6 muscle forces for the adductor magnus and adductor brevis and 27 muscle forces for the other 27 selected muscles. A set of 33 muscle forces were applied to the FE model.

### Geometries of the model

**Fig. 2:**
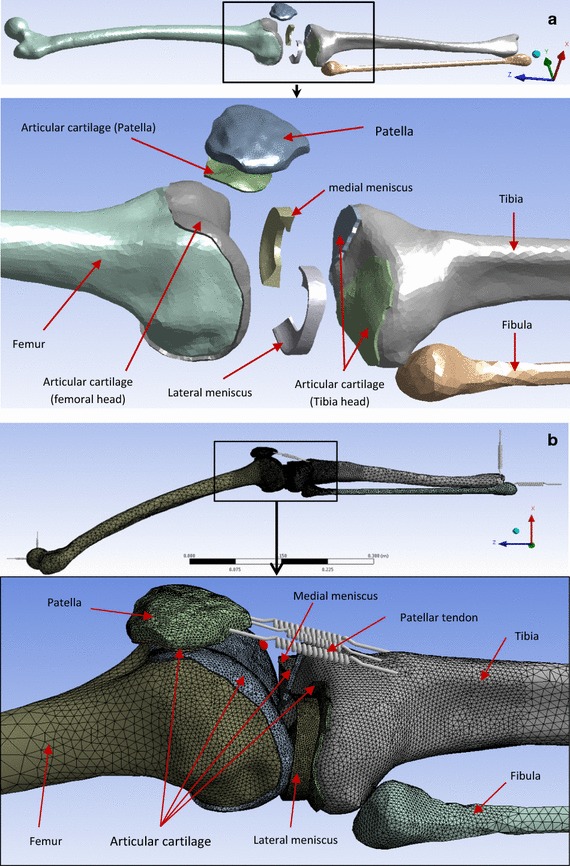
**a** 3D geometries of the parts constituting the FE model of the lower limb. **b** Model mesh: the patellar tendon was modelled with 3 linear springs. The other parts of the model were meshed with tetrahedral solid elements

The FE model was designed using 3D geometries of the femur, tibia, fibula and patella bones and also 3D geometries of the menisci and the articular cartilages present in the knee joint. The 3D geometries of the bones were generated from the mesh of a previous study [29]. This mesh was developed using the state-of-art procedure of 3D geometry acquisition. The data for the procedure were collected using medical computer tomography (CT) scanning and magnetic resonance imaging (MRI) on a subject close to a 50th percentile male [29]. The FE software package HyperWorks-Radioss (Altair Engineering, Inc., Antony, France) was used to generate the geometries of the bones from the existing mesh and to manually create the geometries of the soft tissues based on anatomy books. The geometry data files were then imported into the Design Modeler of the Release 16.2 of ANSYS Workbench FE software package (Ansys, Inc., Canonsburg, Pennsylvania, USA) (Fig. 2a). In order to avoid numerical complexities and keep the model linear, nonlinear contact was excluded in the modelling. Before loading the model, the different parts, bones and soft tissues, were positioned in the selected positions of the stance phase. Penetrations between the parts of the model were avoided during the assembling. All the surface fractions in contact at the interfaces bone–cartilage, menisci-cartilage and femoral cartilage–patellar cartilage were bonded. The ligaments present in the knee joint were not modelled for simplification purposes. The patellar tendon was modelled with three springs.

### Material properties

The material constituting the parts of the model was considered homogeneous, isotropic and linear elastic. To reduce the complexity of the model, the biphasic nature of the soft tissues was not taken into account. Furthermore, considering the short loading time during normal walking compared to the viscoelastic time constant for cartilage, the articular cartilage can be modelled as isotropic linear elastic [4, 20]. The trabecular bone was not modelled in the present study for simplification purposes, thus only the cortical bone was considered. The Young’s modulus of wet embalmed cortical bone of the tibia from younger (41.5 years old) and older (72 years old) men are 18,900 and 16,200 MPa respectively [30]. Hence a Young’s modulus of 17,000 MPa for the cortical bone was considered for the bones. The Young’s modulus of the menisci is higher in the circumferential direction (120 MPa) compared in radial and transversal directions (20 MPa) [20]. Hence a Young’s modulus of 120 MPa was considered to model the menisci as an isotropic linear elastic material. The Young’s modulus was 15 MPa for the articular cartilage [4, 6, 20]. Poisson’s ratio was 0.3 for bones and 0.45 for both soft tissues. The stiffness of the springs modelling the patellar tendon were defined by using the equation

$$k = \frac{E \cdot A}{L},$$

where E was the Young’s modulus, A the surface of the transversal section and L the length of the patellar tendon. The following values were used: E = 900 MPa [31, 32], and A = 160 mm [32, 33]. For the length L of the tendon, a mean value of 5 mm was defined according to the geometry. Hence the stiffness coefficient of the patellar tendon was k = 2880 N/mm, which corresponded to k_spring_ = 960 N/mm for each of the three springs.

#### Application of muscle forces and boundary conditions

All the parts of the model were meshed with 4 node (solid 72) or 10 node (solid 92) solid tetrahedral elements [34] and the patellar tendon was modelled with 3 linear springs as indicated in Fig. 2b. 4 node tetrahedral elements were used in order to reduce the memory size of the model and calculation time.

**Fig. 3:**
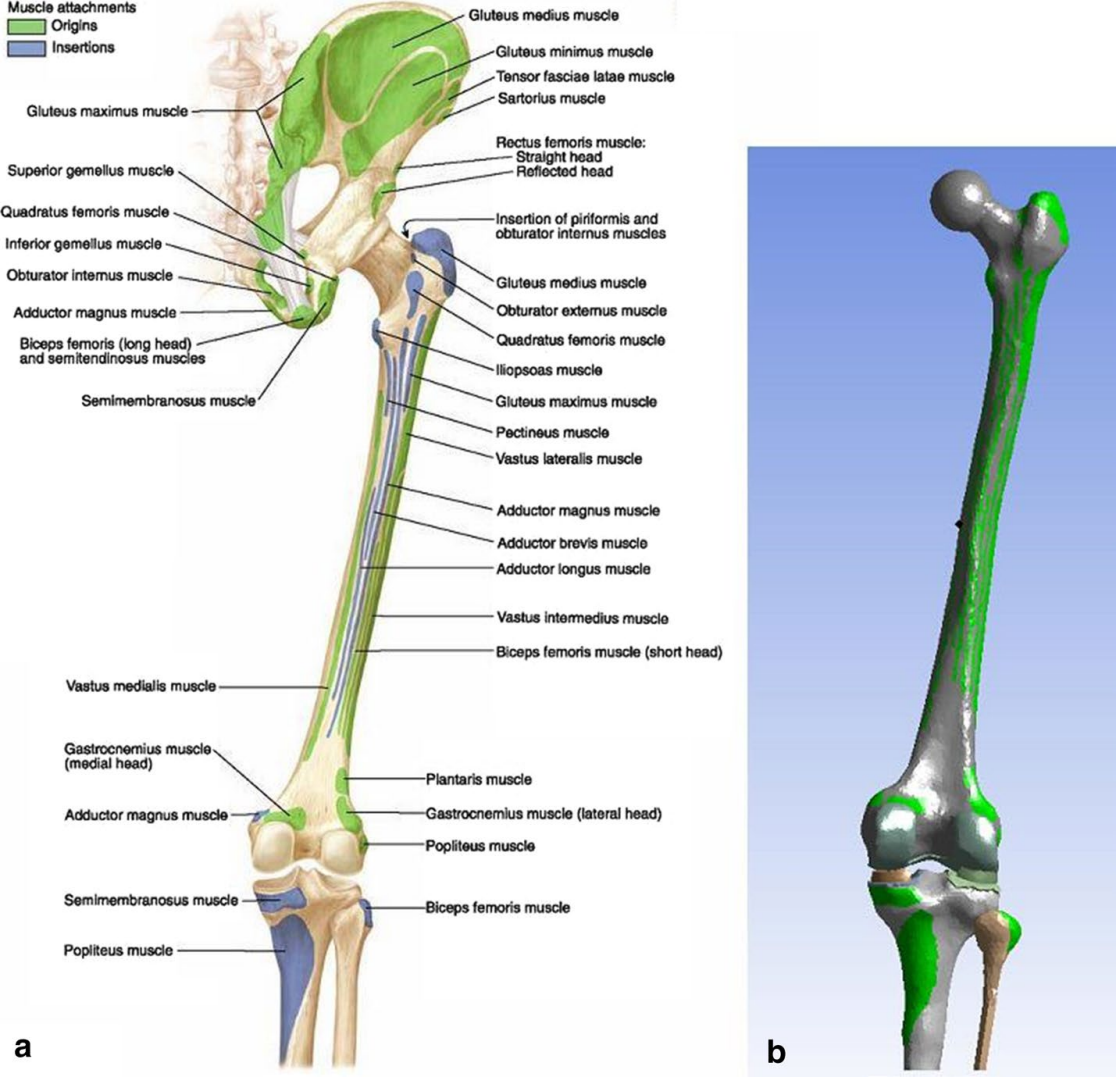
Insertions and origins of muscles included in the modelling: **a** posterior view of anatomical bony attachments of hip and thigh [24]. **b** Reproduction of muscle attachments on the geometry of the FE model

The foot and the leg were taken as a unique segment by considering the ankle joint as rigid. The anatomic muscle attachment areas [35] have been reproduced on bone geometry surfaces in order to apply the corresponding forces of the active muscles (Fig. 3). The law of action–reaction or third Newton’s law was considered to represent the action of any muscle originating and ending on the modelled bones. These muscles were represented by two forces with equal magnitudes but opposite directions applied to the origin and the insertion point.

**Fig. 4:**
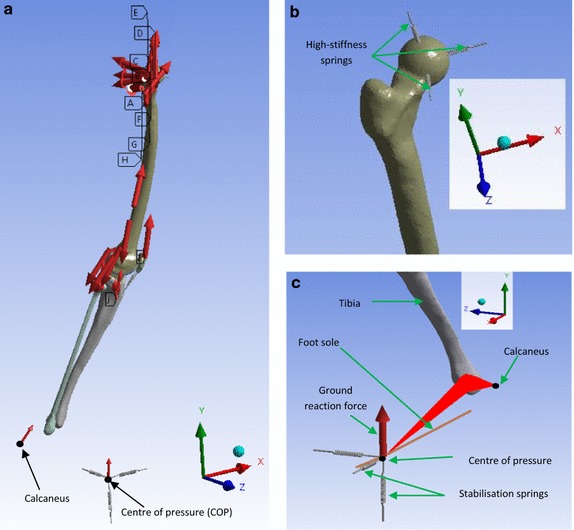
**a** Static analysis of position 4, **b** high-stiffness springs (10^9^ N/mm) fixing the centre of the femoral head to the ground, **c** localisation of the COP and the calcaneus in position 4: the femoral head was fixed to the ground and the GRF was applied to the COP, which was fixed to the ground by means of very weak springs. The COP and the calcaneus were modelled by remote points attached to the distal tibia end. The stabilisation springs are weak springs of 1 N/mm

Since the foot was not included in the model, a segment was used to represent the sole of the foot. This segment was then used to locate the centre of pressure (COP), which is the application point of the GRF. The calcaneus (insertion of the gastrocnemius and the plantaris) and the COP were modelled by remote points (Fig. 4c). The remote points enable the transfer of solicitations to the surface to which they are associated. The geometries of the musculoskeletal RB model and the FE model were derived from two different donors. The measurements for the musculoskeletal model (AnyBody version 6.0) were performed on the right lower extremity of a male (age 77, height 1.74 m, weight 105 kg). The geometry was then scaled to the dimensions of the person (height 1.62 m, weight 62 kg) whose normal gait was considered. The bone geometries for the FE mesh, as already stated, were derived from CT and MRI scans collected on a subject close to a 50th percentile male. Therefore, to make sure that the two models in the two systems were aligned, for each of the 5 selected positions, the geometry of the FE model was modified and positioned, at the same corresponding position of the musculoskeletal RB model. The positioning was made firstly by choosing the following anatomical markers of the musculoskeletal RB model: the centre of the femoral head, lateral and medial femoral epicondyles, and medial malleoli. Secondly, the following three points of the femur of the FE model were then selected: the centre of the femoral head, the middle of the transepicondylar axis and the medial epicondyle. Then the selected three points were positioned, so that they coincided with the corresponding three markers of the femur of the musculoskeletal RB model [36]. The tibia and the two menisci were then positioned, so that the menisci were in contact with the articular cartilages of the distal femoral head and the tibia head while avoiding interpenetrations. However due to the difference of the form of the two tibiae the malleoli of the two models were not perfectly aligned, though the angle formed by the two tibia axes in the frontal plane was less than 3°. This appeared acceptable and the components of the predicted muscle forces were applied as external load to the FE models in any selected position. The muscle forces were modelled as distributed load over the muscle attachment area.

Due to the fact that the patellar cartilage was bonded to the femoral cartilage, the quadriceps force was partly transferred to the femur instead of the tibia. However, as Young’s modulus of cartilage is quite small, the connection between the patellar cartilage and the femoral cartilage was soft and the transmitted shear forces were small. According to Saint–Venant’s principle, this influences the stress distribution only at that interface and does not affect the stress distribution at more distant locations. Three stabilisation springs with weak stiffness of 1 N/mm and oriented in the x, y and z-direction were attached at the distal basis of the tibia in order to avoid numeric instability of the model (Fig. 4a, c). The three translational degrees of freedom of the femur were constrained by using a spherical joint realised by fixing the centre of the femoral head to the ground with three springs. These three springs had a high stiffness (10^9^ N/mm) and were oriented in the three directions of space (Fig. 4a, b).

### FE analyses and validation of the models

The analyses were performed using ANSYS Workbench (Ansys, Inc., Canonsburg, Pennsylvania, USA). For any of the five selected positions of the stance phase of the gait, the following displacements and forces were calculated: the displacements of the distal end of the tibia relative to its initial position prior to the application of the muscle actions, the forces in the stabilisation springs, and the reaction forces at the femoral head. Table 2 recapitulates the muscles included in the FE models and the magnitudes of the GRF for each position. A model was considered as valid when: (1) the deformations resulting from the applied loads were such that the displacement of the distal end of the tibia was nearly zero, consistent with Newton’s first law; (2) the magnitudes of the forces in the stabilisation springs were negligible and (3) the reaction forces at the femoral head were similar to the predicted hip joint forces of the musculoskeletal RB model. The strains were checked to stay within a reasonable range.

**Table 2:** Magnitudes of the muscle forces and the GRF at each position

Muscles	Position 1 (16 muscles)	Position 2 (19 muscles)	Position 3 (19 muscles)	Position 4 (25 muscles)	Position 5 (19 muscles)
AdductorBrevisDistal	√			√	√
AdductorBrevisMid	√			√	√
AdductorBrevisProximal	√			√	√
AdductorLongus	√			√	√
AdductorMagnusDistal	√	√			√
AdductorMagnusMid	√	√			
AdductorMagnusProximal	√				√
BicepsFemorisCaputBreve (×2)	√				√
BicepsFemorisCaputLongum	√	√			
GastrocnemiusMedialis (×2)			√	√	√
GastrocnemiusLateralis (×2)			√	√	
GemellusInferior		√	√	√	√
GemellusSuperior		√	√	√	
GluteusMaximus	√	√			
GluteusMedius		√	√	√	
GluteusMinimus		√	√	√	
Gracilis	√			√	√
Iliacus			√	√	√
ObturatorExternus	√	√	√	√	√
ObturatorInternus		√	√	√	√
Pectineus	√			√	√
Piriformis		√	√	√	√
Plantaris (×2)			√	√	
Popliteus		√	√	√	
QuadratusFemoris	√	√	√	√	√
RectusFemoris		√	√	√	√
Sartorius			√	√	√
Semimembranosus	√	√			
Semitendinosus	√	√			
TensorFasciaeLatae			√	√	√
VastusIntermedius (×2)		√	√	√	
VastusLateralis (×2)		√	√	√	
VastusMedialis (×2)		√	√	√	
Components of the GRF [N]	253	592	483	644	15

(x2) means that the muscle action was modelled by two opposite forces with equal magnitude. √ means that the action of the muscle was applied for the selected position

## Results

**Fig. 5:**
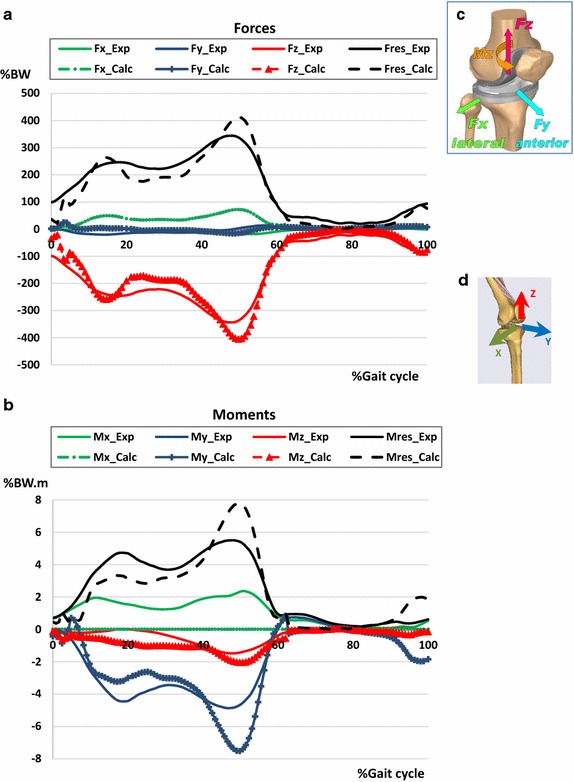
Comparison of the experimental and calculated knee contact forces applied on the tibia plateau during normal walking: **a** forces. **b** Moments. **c** Reference system used to define the experimental forces retrieved from the database OrthoLoad [18]. **d** Reference system used to define the calculated knee joint forces by means of the musculoskeletal model “GaitFullBody” from the model repository in the software AnyBody. The indices “calc” and “exp” in the legends refer to the calculated and the experimental quantities respectively. The knee joint is modelled as a hinge joint anchored at the middle of the femoral transepicondylar axis. The X axis is the axis of the hinge joint hence the component Mx is equal to zero

Figure 5 shows the plots of the predicted and measured knee joint contact forces. The measured contact forces, already published elsewhere [12], are presented here for the sake of comparison. The difference observed for the components Fx_calc, Fx_exp can be related to the fact that the knee joint of the musculoskeletal RB model was modelled as a revolute joint, which does not allow translations and provides a single-axis rotation around the x-axis. This is the reason why the calculated moment Mx_calc about the x-axis was equal to zero. The components of the force in the postero-anterior direction (Fz_calc, Fz_exp) were negligible compared to the vertical components of the force. The vertical components of the force (Fy_calc, Fy_exp) and the resultant forces (Fres_calc, Fres_exp) were qualitatively similar. The same observation is valid for the moments about the vertical axis (My_calc, My_exp and the resultant moments (Mres_calc, Mres_exp). The values of the predicted resultant forces were 261% BW at the first peak and 412% BW at the second. The RMS errors between the resultant force and moment were 35.75% BW and 1.01% BW m respectively.

**Fig. 6:**
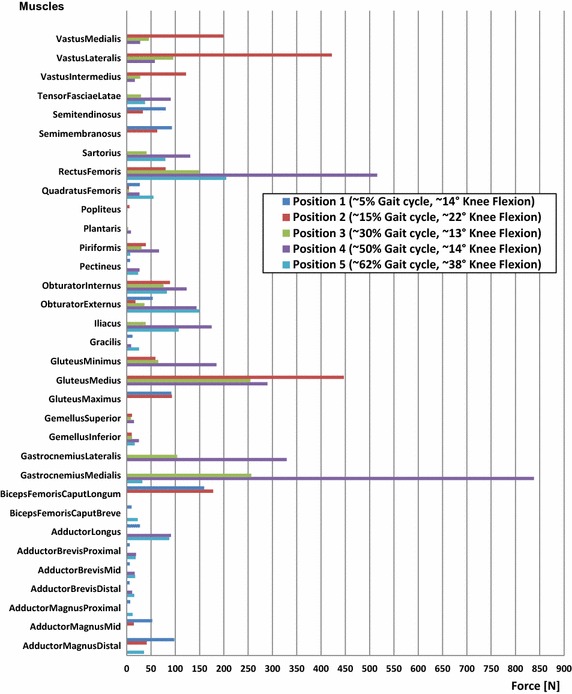
Magnitudes of the forces of the activated muscles at the 5 selected positions. The sets of the active muscles are different from one position to another

The sets of the active muscles are different from one position to another. The figure below (Fig. 6) shows the magnitudes of the selected active muscles for each position. The muscle forces’ magnitudes were highest at position 4 (50% Gait cycle, ~ 14° knee flexion), which corresponded to the start of the propulsion phase, when the foot pushed off the ground to propel the body forward.

The reaction forces at the femoral head and the forces of the stabilisation springs are summarized in Table 3. The reaction forces at the femoral head correspond to the hip joint reaction forces calculated with the RB model.

**Table 3:** Reaction forces at the femoral head and small weak spring forces resulting from the applied muscles forces and the GRF

Positions	Forces	Force components [N]			Fres [N]
		Fx	Fy	Fz	
Position 1	Reaction at femoral head	432	− 747	130	873
	Action of the stabilisation springs	− 11	− 5	− 2	13
Position 2	Reaction at femoral head	364	− 1477	370	1566
	Action of the stabilisation springs	1	− 3	17	18
Position 3	Reaction at femoral head	45	− 1017	272	1054
	Action of the stabilisation springs	− 16	− 3	11	20
Position 4	Reaction at femoral head	− 580	− 1935	552	2095
	Action of the stabilisation springs	− 27	2	22	34
Position 5	Reaction at femoral head	− 176	− 530	384	678
	Action of the stabilisation springs	− 6	2	7	10

The reaction forces at the femoral head correspond to the hip reaction force. Fres is the resultant force

The highest force magnitude of the stabilisation springs (34 N) was obtained in position 4 at 50% of the gait cycle. At this moment, the knee flexion was around 14° and the magnitude of the hip joint reaction force was at its highest (2095 N). The action of the stabilisation springs was smallest when the lower limb was in position 5 (62% gait cycle and 38° knee flexion). The hip joint reaction force was also the smallest (678 N) in position 5.

Table 4 summarises the largest deformations, i.e. the displacements of the model that results from the muscle actions on the FE models of the lower limb in the 5 selected positions. The model rotated around the centre of the femoral head.

**Table 4:** Maximal deformations of the model in the different selected positions

Positions	Components of the deformations [mm]			Total deformations [mm]
	Dx	Dy	Dz	
Position 1	10.7	5.3	5.1	12.2
Position 2	1.6	3.4	3.2	16.2
Position 3	12.3	3.2	1.3	19
Position 4	32.4	6.4	− 11.1	50
Position 5	8.4	0.7	− 3.8	17.3

**Fig. 7:**
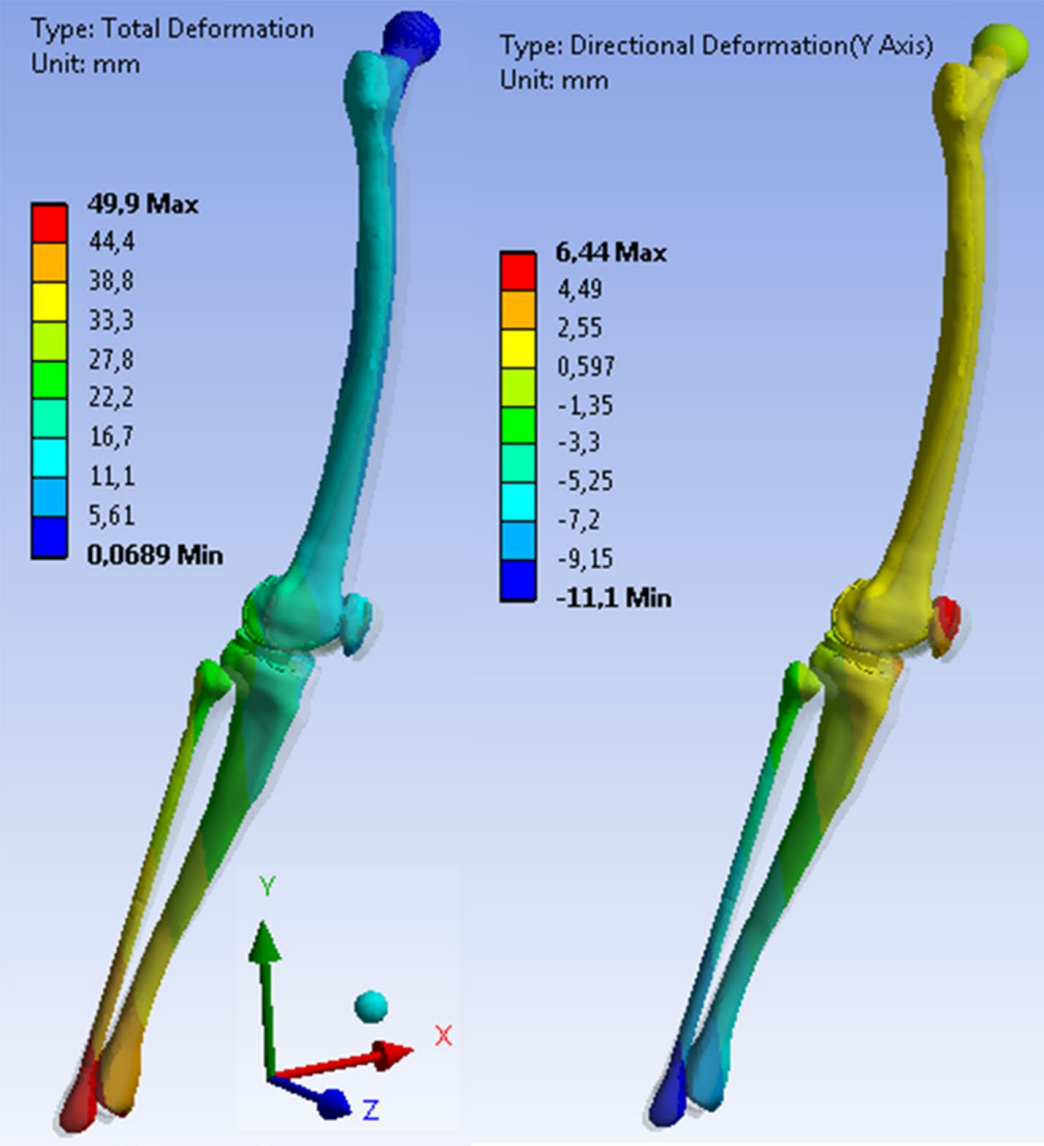
Total deformation and deformation in the distal–proximal direction (y) of the model in position 4

The largest deformation of the model was the displacement of the distal end of the tibia; 12.2 mm in position 1, 16.2 mm in position 2,19 mm in position 3, 50 mm in position 4 (Fig. 7) and 17.3 mm in position 5. This deformation resulted from the translation due to the elastic strain and rigid body rotation around the femoral head. This is shown by the values of the displacements (Dx and Dz) of the distal part of the tibia in the horizontal plane, which was higher than the component (Dy) in the vertical direction (Table 4).

Table 5 compares the hip joint forces from the musculoskeletal RB model to the hip joint reaction forces of the present FE model.

**Table 5:** Inertial and reaction forces

Position	Inertial forces ma [N]		Hip joint reaction forces [N]			Forces of the stabilisation springs [N]
	Thigh	Leg and foot	RB model (N)	FE model (N)	Relative difference (%)	
Position 1	26	22	765	873	14	13
Position 2	15	43	1498	1566	4.5	18
Position 3	12	7	998	1054	5.6	20
Position 4	16	18	2077	2095	0.8	34
Position 5	17	32	586	678	15.7	10

The soft spring forces and the inertial forces are considered negligible compared to the hip joint reaction forces. The relative difference was estimated by considering the magnitude of the hip joint reaction forces from the RB model as reference value

The inertial forces and the forces of the stabilisation springs had similar magnitudes and were considered negligible compared to the hip joint reaction forces. The hip joint reaction forces from the RB model were smaller than those from the FE model, but nevertheless the two reaction forces were similar. The relative differences obtained by applying the muscle forces from the RB model to the FE models were less than 16%. The smallest relative difference (0.8%) was obtained for the lower limb in position 4 and the highest (15.7%) in position 5 (Table 5).

**Fig. 8:**
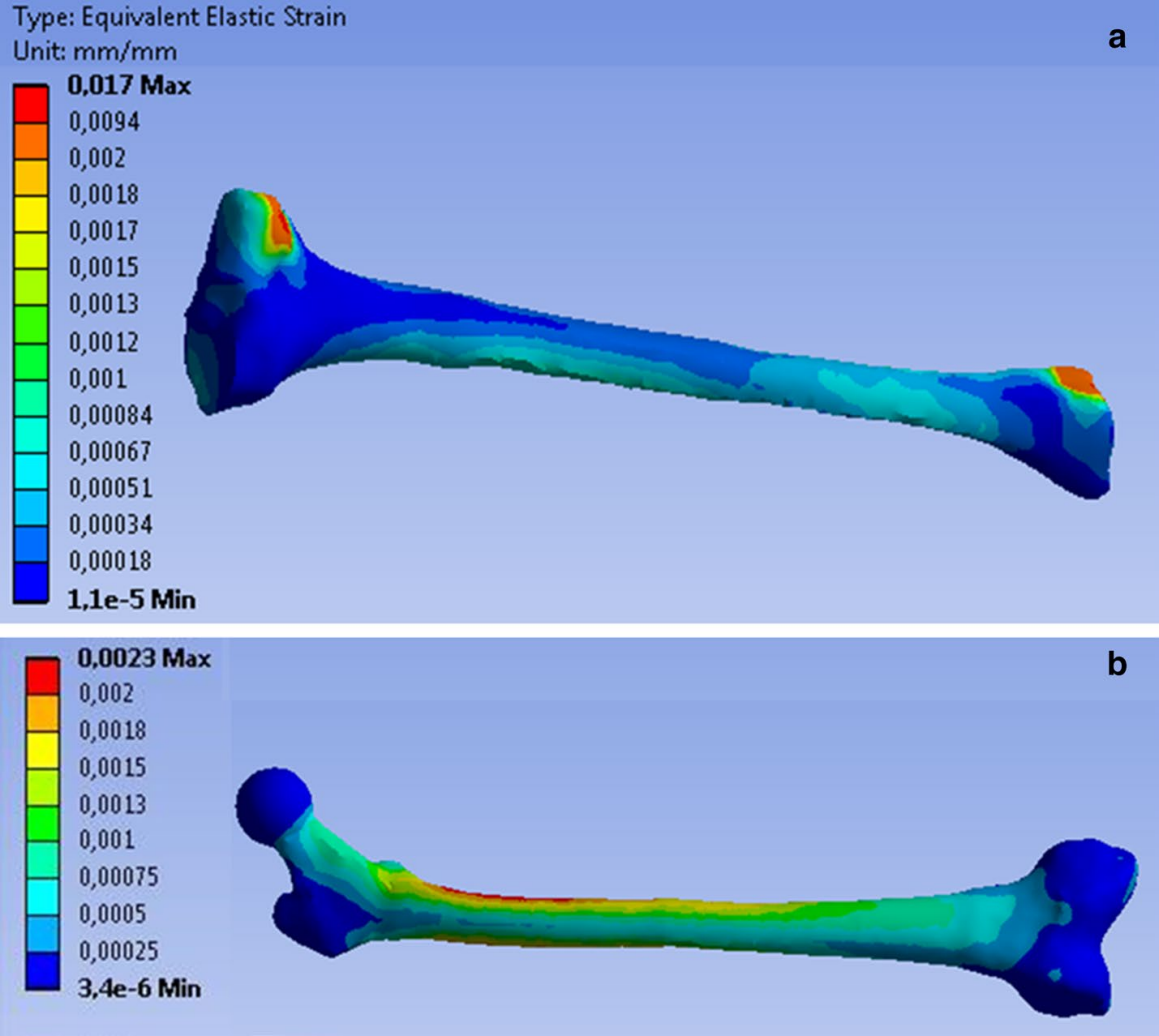
Elastic strain of tibia **a** and femur **b** for the lower limb in position 4. The high value (0.017) is due to the bonded contact between the tibia and the fibula. The highest strain of 0.0023 = 2.3‰ is located in the proximal region of the diaphysis

Maximal strains were obtained when the lower limb was in position 4. In the tibia, the highest value was 1.7% and was located in the contact zone between the tibia and the fibula (Fig. 8a). The highest strain value in the femur was 0.23% and was located in the proximal region of the diaphysis (Fig. 8b). This confirms the fact that the displacement of the distal end of the tibia was mainly due to rigid rotations of the model around the centre of the femoral head.

Considering that: (1) the displacements of the COP that corresponds to the displacements of the distal end of the tibia were negligible; (2) the actions of the stabilisation springs were insignificantly small relative to the GRF and the reactions force at femoral head and (3) the reaction forces at the centre of the femoral head were similar to the hip joint reaction forces from the musculoskeletal RB model, the current models at the five selected positions of the stance phase of gait can be considered to be valid.

## Discussion

The overall objective of this study was to present a FE model of the lower limb considering the muscle forces in a detailed manner. The selected muscle forces reflect the stance phase of the gait and were calculated by a validated musculoskeletal RB model of the human body that is present in the repository of the musculoskeletal modelling software AnyBody [13–15, 23]. The predicted knee joint forces by means of the used musculoskeletal RB model were similar to the measured knee contact forces from the works of Bergmann et al. (file K7L_280710_1_28P of the patient K7L from database OrthoLoad) [12]. The quantitative differences of the moments observed can be related to the fact that experimentally measured moments were defined in a coordinate system with its origin located on the plateau of the knee prosthesis that contained the telemetric instrumentation, while the calculated moments were defined in a coordinate system with its origin located on the transepicondylar axis. The RMS error between the resultant forces was 35.75% BW, which is less than the maximal difference of 100% BW between the resultants of the knee joint forces measured during walking in five different subjects reported by Kutzner et al. [11]. The predicted muscle forces had then been applied to the geometry of a FE model of the lower extremity. The muscle forces were modelled as distributed over the muscle attachment area. It was shown that the reaction forces at the centre of the femoral head were similar to the hip joint reaction forces from the musculoskeletal RB model. Additionally, the action of the stabilisation springs that were attached at the COP was negligible, thus allowing us to consider the FE model as valid, as Newton’s first law was satisfied.

The actions of the muscle forces on bony structures are more realistic in the present model as the muscle forces were distributed over their attachment areas. Polgar et al. [38, 39] demonstrated that applying muscle forces as concentrated loads at the centroids of their attachments may lead to unrealistic results. The distribution of strains in the femur (Fig. 8b) was similar to the estimated strain in the preceding study of Duda et al. [40]. They reported maximal values of the strain on the medial proximal femur (2000 με = 0.002) under physiological loading taking into account the muscle forces during the stance phase of the gait. Venäläinen et al. [41] reported strain values up to 0.05% in homogeneous tibia under loading conditions corresponding to the first 20% of stance. These values matched the strain distribution in tibia obtained in the present study, since values above 0.05% until 0.17% were due to contact between fibula and tibia (Fig. 8a). Venäläinen et al. did not consider the fibula in their study [41].

Sun et al. [42] simulated two positions of the knee joint in order to analyse the stress distribution on the tibia plateau. The two positions corresponded to the two peak values of GRF. Sun and his colleagues fixed the proximal end of the femur and the peak values of the vertical component of GRF were applied to the distal end of the tibia and fibula. The study by Sun et al. was limited to the sagittal plane, ignoring the effect of the transversal component in the frontal plane of GRF, which contributes together with the vertical component to the knee abduction moment [43]. The present study considered all components of GRF for the loading conditions of the FE model.

Adouni et al. [21, 22] developed a kinematics-driven musculoskeletal FE model in order to investigate contact pressure within the knee joint while predicting the muscle forces during the stance phase of gait. They simulated contact interfaces as frictionless contact with no penetration [44, 45], however the bony structures were rigid. Our models that are presented in this study considered deformable bones, but the contact interfaces were bonded in order to keep the model linear and avoid convergence problems. This represents a limitation of the present study that should be overcome if one would like to investigate the stresses/strains of the cartilages and menisci, additional to the performance of osteotomy fixation devices, which result from more realistic and accurate loading conditions.

Kiapour and colleagues [19, 20] developed FE models which incorporated models of all the soft tissues within the knee joint, but only parts of the bony structures were considered, namely the proximal femur, the distal tibia and the distal fibula. Their models aimed to be used in the clinical evaluation of risk factors associated with anterior cruciate ligaments injury and were validated against data measured from static, quasi-static and dynamic cadaveric experiments. In order to validate the model, the loading conditions were restricted to knee abduction and internal tibia rotation moments, anterior tibia shear and simulated muscle loads of the quadriceps and the hamstrings. Muscle forces responsible for the motions that cause tissue injuries should have been considered in more detail, as we did in our study.

We decided to neglect the gravity action on the models as the centre of the femoral head was attached to the ground and the GRF was applied to the distal part of the tibia. This also had the advantage of considerably reducing the computation time. The inertial forces were not considered in the model because they are not significant during the stance phase of gait, as shown in the present study (Table 1) and by other authors [46]. These simplifications explain the differences observed between the values of the hip joint reaction forces from RB model and from the FE model (Table 5). These differences were smaller than 16% and considered negligible. The present FE model cannot directly be validated against experimental data, but the muscles forces that were applied to the model were extracted from a musculoskeletal RB model of the lower limb that was validated against experimentally measured joint contact forces [16, 17]. In order to reduce the complexity of modelling, the trabecular bone was not modelled, the bones and the soft tissues were considered as linear isotropic and the contact interfaces between the parts as bonded. The fact that ligaments of the knee joint were not modelled constitutes another limitation of the present FE model. Those considerations may disqualify the present model for the analysis of strains and stresses of the soft tissues within the knee, but the model can be used for the analysis and the design of knee implants under consideration of more realistic physiological loading during the stance phase. To achieve this aim further works consisting in including the knee joint implant geometries and correct implant contact mechanics would be required. One direct application of high relevance is the analysis of HTO implants. The model can be used to predict stresses and strains in HTO plates.

## Conclusions

The approach considered for the present FE modelling can be used to perform analyses of the lower limb taking into account realistic boundary conditions. This approach will lead to results that give better insight into the biomechanics of the knee joint. The model can be readapted depending on the objectives of the study of the knee joint. In the present form, this model can be used to study the performances of osteotomy fixation devices.

## Abbreviations

FE: finite element
HTO: high tibial osteotomy
RB: rigid body
GRF: ground reaction force
CT: computer tomography
MRI: magnetic resonance imaging
COP: centre of pressure
